# Assessment of Performance, Microbial Community, Bacterial Food Quality, and Gene Expression of Whiteleg Shrimp (*Litopenaeus vannamei*) Reared under Different Density Biofloc Systems

**DOI:** 10.1155/2022/3499061

**Published:** 2022-09-21

**Authors:** Mohamed M. Said, Y. A. El-barbary, O. M. Ahmed

**Affiliations:** ^1^Department of Aquaculture, Faculty of Fish Resources, Suez University, Suez, Egypt; ^2^Department of Fish Health and Diseases, Faculty of Fish Resources, Suez University, Suez, Egypt; ^3^Department of Fish Processing and Technology, Faculty of Fish Resources, Suez University, Suez, Egypt

## Abstract

Biofloc shrimp culture, as a way of improving shrimp production, gains worldwide consideration. However, the effects of the biofloc system on shrimp culture at high densities could be a challenge. Here, this study is aimed at identifying a better stocking density of whiteleg shrimp (*Litopenaeus vannamei*) between two intensive biofloc systems of 100 and 300 org./m^2^. Achieving that was done by comparing growth performance, water quality, feed utilization, microbial loads from water and shrimps, and gene expression of growth, stress, and immune-related genes. Shrimp postlarvae with a mean weight of 35.4 ± 3.7 mg were reared in six indoor cement tanks (36 m^3^ total capacity each) at two stocking densities (3 replicates each) for a rearing period of 135 days. Better final weight, weight gain, average daily weight gain, specific growth rate, biomass increase percentage, and survival rate were associated with lower density (100/m^2^), whereas high-density showed significantly higher total biomass. Better feed utilization was found in the lower density treatment. Lower density treatment enhanced water quality parameters, including higher dissolved oxygen and lower nitrogenous wastes. Heterotrophic bacterial count in water samples was recorded as 5.28 ± 0.15 and 5.11 ± 0.28 log CFU/ml from the high- and low-density systems, respectively, with no significant difference. Beneficial bacteria such as *Bacillus* spp. were identified in water samples from both systems, still, the *Vibrio*-like count was developed in the higher density system. Regarding shrimp food bacterial quality, the total bacterial count in the shrimp was recorded as 5.09 ± 0.1 log CFU/g in the 300 org./m^2^ treatment compared to 4.75 ± 0.24 log CFU/g in the lower density. *Escherichia coli* was isolated from the shrimps in a lower density group while *Aeromonas hydrophila* and *Citrobacter freundii* were associated with shrimps from a higher density system. Immune-related genes including prophenoloxidase, superoxide dismutase (SOD), and lysozyme (LYZ) expressions were all significantly higher expressed in the shrimp from the lower density treatment. Toll receptor (LvToll), penaiedin4 (PEN4), and stress-related gene (HSP 70) showed a decreased gene expression in the shrimp raised in the lower density. **S**ignificant upregulation of growth-related gene (Ras-related protein-RAP) expression was associated with the lower stocking density system. In conclusion, the current study found that applying high stocking density (300 org./m^2^) contributes negatively to performance, water quality, microbial community, bacterial food quality, and gene expression of immune, stress, and growth-related genes when compared with the lower stocking density system (100 org./m^2^) under biofloc system.

## 1. Introduction

Intensive shrimp culture represents one of the promising ways of improving aquaculture production [1]. Biofloc technology is a sustainable technique for intensive shrimp production in many ways. It improves water quality by transferring organic nitrogenous waste or ammonium into bacterial biomass, which is used as an added source of proteinaceous feed [2]. Bacterial populations in biofloc technology (BFT) were mainly responsible for water quality preservation in minimal or zero water exchange systems [3, 4]. The Biofloc system enhanced the growth of beneficial heterotrophic bacteria which in return improved the survival, growth, and immunity of the shrimp production [3, 5, 6].

Advantages of the biofloc system include aggregates of beneficial microorganisms which improve shrimp microbiota and food bacterial quality through synthesizing antimicrobial compounds [1, 7, 8]. In contrast, bacteria such as *Vibrio* spp. are considered a challenge in aquaculture. Seawater is considered their natural habitat and could establish about 40% of the bacterial community [9]. They are also a portion of the natural microflora of crustacea, fish, and shellfish [10]. Some species of *vibrios* including *V. vulnificus* and *V. parahaemolyticus* are pathogenic and may cause seafood-borne illnesses in humans [11–13]. Seafood-borne outbreaks caused by *vibrios* in humans have been reported worldwide [14, 15].

Biofloc can also act as immuno-stimulants to improve the shrimp's immune system [16]. It was proved that biofloc microorganisms also suppress pathogen growth by competing for space, substrate, and nutrients and by excreting inhibiting compounds. Biofloc has probiotic effects, thus crowding stress on the culture organisms may be reduced or even eliminated [17].

The benefits of the biofloc system could be challenged by inappropriate stocking density. Unrestrained high stocking density had a negative role in shrimp production. It induced declined water quality and crowding, which lead to physiological changes resulting in lower feed utilization efficiency and growth performance [18–21]. Some studies reported negative effects of high densities on whiteleg shrimp under the biofloc system. These findings included survival and growth of larval and postlarval stages [22], nursery phase and stress resistance [23] immunities, antioxidant status, and resistance against *Vibrio* [17].

This study is aimed at investigating growth performance, water quality, feed utilization, microbial loads from water and shrimps, and gene expression of growth, stress, and immune-related genes of shrimps reared under a biofloc system with a stocking density of 300 org./m^2^ as compared with the lower density (100 org./m^2^) to determine the possibility of applying biofloc system for overcoming some constraints of the shrimp ultraintensive production.

## 2. Materials and Methods

### 2.1. Location and Duration

The experiment lasted for 135 days at a marine shrimp hatchery located in Damietta, Egypt during the period from June to October of 2021. The experiment was done in six cement tanks with a volume of 36 m^3^ total capacity each. The experimental tank's dimensions in meters were 3 (width) ∗10 (length) ∗ 1.2 (depth). Each tank is filled with 30 m^3^ of sand-filtered seawater (30 ± 0.5 ppt).

### 2.2. Experimental Design

Postlarvae of *L. vannamei* shrimp with a mean body weight of 35.4 ± 3.7 mg. were obtained from a private shrimp hatchery and stocked at two different stocking densities (100 and 300 shrimp/m^2^) with triplicate for each treatment.

### 2.3. Experimental Conditions

Tanks were all provided with nonstop aeration and 12 h dark and 12 h light regime. Four air pumps with 5.5 horsepower were used to supply the aeration network which starts with distribution pipes (2 inches), and each pipe is attached to a regulator to control the air pressure in all tanks. To aerate and mix the culture water in all tanks, a web of air stones was installed at the bottom of each tank.

Wheat flour was added to all experimental units as a source of carbon to promote the development of bioflocs. The carbon source was added once a day [24]. Input C : N ratio was maintained as 15 : 1 by adding the required weight of the carbon source. The preweighed carbon source was mixed with cultured water and equally spread over the tank's surface [25].

### 2.4. Feeding Management

Shrimps were fed 4 times/day at 8 AM, 11 AM, 2 PM, and 4 PM with commercial shrimp feed 38% protein (Skretting, Egypt). The composition of carbohydrate source and shrimp feed is shown in Table 1. Daily feeding rates gradually decreased from 15% to 2% of the shrimp's body weight throughout the whole experiment time. The amount of feed was recalculated every two weeks after weighing representative shrimp samples from each tank. Feed was introduced to the shrimps as crumbles (0.4-0.6 mm) during the first three weeks, and then pelleted feeds with diameter increased from 0.8 to 1.5 mm till the terminal of the experiment.

### 2.5. Water Quality Monitoring

Dissolved oxygen (DO), temperature, ammonia, nitrite, turbidity, and biofloc volume were all monitored throughout the experiment. The water profile at the start of the experiment was as follow: water temperature = 27.5 ± 0.3°C, pH = 7.1 ± 0.2, and DO = 5.3 ± 0.2 mg/L. An electronic probe was used to measure the dissolved oxygen and water temperature (HANNA, HI9146-04). Ammonia and nitrite were measured using a photometer (HANNA, HI97715), while portable pH meter (Milwaukee, MW102) was used to measure the pH of water. Turbidity was monitored with a turbidity meter (Lovibond, TB211 IR) while floc volume was measured using an Imhoff cone.

### 2.6. Growth Performance and Survival Rate

The shrimps' weights were checked on two weeks basis to follow up on the growth and adjust the amount of feed and organic carbon addition. The weights were measured using an electronic balance. Shrimps were collected at the experiment termination after draining the tanks. The remaining number of shrimps at the terminal of the experiment was recorded to calculate the survival rate in each tank. Weight gain (WG), final weight (FW), average daily weight gain (ADWG), specific growth rate% (SGR%), final biomass, and percentage of biomass increase were all measured to compare the growth performance between the two different stocking densities. Feed utilization performance was represented as feed efficiency (FE), feed conversion ratio (FCR), and protein efficiency ratio (PER). Growth, feed utilization and survival rate were determined based on the following equations: body weight gain (g) = final weight − initial weight. Average daily weight gain (ADWG) = (final weight − initial weight)/days of the experiment. Specific growth rate (SGR) = (natural logarithm (Ln) final weight − Ln initial weight)/days of experiment. Biomass = (Final weight∗harvested number of shrimps). Biomass increase percentage = (final number∗final weight)/(initial number∗initial weight)∗100. Feed conversion ratio (FCR) = feed consumed (dry weight)/live weight gain (wet weight). Feed efficiency (FE) = (final weight − initial weight)/feed consumed∗100. Protein efficiency ratio (PER) = net weight gain (wet weight) (g)/protein consumed (g). Survival rate (SR) = (number of individuals at the end of experiment/initial number of individuals stocked)∗100 [26].

### 2.7. Bacteriological Analysis

Water samples (100 ml) and 60 random shrimps were selected separately from each experimental tank (10 from each tank) at the terminal of the experiment period. Samples were kept in sterile bags and placed in a cool polystyrene box containing sterile ice packs that kept the temperatures at 4-6°C during transportation [27]. Samples were cautiously transported to the Faculty of Fish Resources laboratories, Suez University, and were analyzed instantly.

#### 2.7.1. Sample Preparation

Shrimp samples were beheaded, chopped into small pieces aseptically, and placed on a sterile tray. Individual shrimp samples were homogenized with 45 ml of buffered peptone water (0.1%) (Lab M, UK) for 2 min. using a stomacher (Seward Stomacher 400 circulator, UK), while water samples (1 ml) were vortexed for 2 min. in a sterile falcon tube with 9 ml of buffered peptone water (0.1%) [27].

#### 2.7.2. Heterotrophic Bacterial Count

Serial dilution to tenth folds was done for the total bacterial count. Dilutions up to 10^5^ were spread onto a plate count agar (PCA, Oxoid, UK). According to FDA [27] and the bacterial count was reported as a log of colony-forming units (log CFU/g) for shrimp samples and (log CFU/ml) for water samples. The experiments were done in duplicates and the results were demonstrated as means ± standard deviations.

Colonies of different morphology were selected from the plate count agar and inoculated on the Trypticase soy agar (TSA, Lab M, UK) slant. Selected bacterial colonies were Gram-stained and then identified biochemically with indole, Voges-Proskauer (VP), and methyl red tests [27]. *Bacillus* spp. was characterized as gray-white round on nutrient agar, Gram-positive bacillus, positive for VP test, and negative for indole and methyl red [28]. Other bacterial species were identified using previous biochemical tests and confirmed with API 20E strips (BioMérieux, France) [29, 30]. Procedures for using API 20E strips and bacterial identification were done according to the manufacturer's instructions.

#### 2.7.3. *Vibrio* Count

The shrimps (5 g) were homogenized with alkaline peptone water (45 ml) (lab M, UK) containing 1% NaCl for 2 min by a stomacher (Seward 400 circulator, UK). Likewise, water (1 ml) was vortexed in alkaline peptone water (9 ml), and then incubated at 35 ± 2°C for 24 h. Enrichment cultures were platted onto thiosulphate-citrate-bile salts-sucrose (TCBS- lab M, UK), where yellow and green colonies were counted as *Vibrio*-like colonies. Selected colonies were streaked onto TSA slants supplemented with 1% NaCl. After incubation at 35 ± 2°C/24 h, the isolates were tested biochemically with oxidase test and API 20E diagnostic strips (BioMérieux, France) [31, 32].

### 2.8. Total RNA Extraction, cDNA Synthesis, and Gene Expression Analysis by Quantitative Real-Time PCR

The operating protocol for this analysis was in running order according to the methods shown by Aguilera-Rivera et al.*, [*33*]*. Concisely, total RNA was isolated from hemolymph (the volume of samples was not exceeded 10% of the used Trizol volume) from freshly collected shrimp by instructions of the Trizol reagent. The isolated RNA was measured at 260 and 280 nm with a spectrophotometer (UNICO-UV-VIS Spectrophotometer). RevertAid First Strand cDNA Synthesis Kit® (Thermo Scientific) was used for the efficient synthesis of cDNA from RNA templates according to kit instructions.

Specific primers (METBION®) for immune-related genes were selected to perform quantitative real-time PCR (RT-qPCR) based on previous published *L. vannamei* primer sequences for the following genes: prophenoloxidase, superoxide dismutase, Toll receptor, penaeidin4 and lysozyme (immuno-related genes), heat shock protein-70 (Lvhsp70) (stress-related genes), Ras-related protein (Rap-2a) (growth-related genes), and *β*-actin gene (internal control) (Table 2). The RT-qPCR was performed by 7500 Fast Real-Time PCR System® Applied Biosystem using SYBR Green master mix (Top Real SYBR mix®, Biovision).

Sample duplication was operated for each sample. RT-qPCR cycle set consisted of 15 min of initial denaturation at 95°C, then 40 thermal cycles at 95 °C for 15 s, followed by 60°C for 30 s along with 1 min at 60°C. The transcriptional regulation of the immune, stress, and growth-related shrimp genes was evaluated using an RT-qPCR assessment. Following the 2^−ΔΔct^ equation, the data have been presented as the fold-change in expression levels of the target gene customized to an internal reference gene (*β*-actin) and in proportion to the control (high-density group) [34].

### 2.9. Statistical Analysis

Data were statistically analyzed using IBM SPSS Statistics version 25 (IBM Corporation, NY, USA). An independent sample *t*-test was used to examine the effects of different stocking densities on growth performance, feed utilization, survival rate, bacterial counts, and gene expression. Parameters of water quality were related by two-way repeated-measures ANOVA with treatment as the key aspect and sampling date as the repeated measures factor. Results were expressed as the mean ± SD. Mean differences were compared by Duncan's multiple range tests. A probability value (*P*) of less than 0.05 was used to indicate statistically significant differences.

## 3. Results and Discussion

### 3.1. Growth Performance, Feed Utilization, and Water Quality

Lower density treatment showed a positive impact on *L. vannamei* growth performance. Final weight, weight gain, daily weight gain, specific growth rate, biomass increase percentage, and survival rate were all significantly higher (*P* < 0.05) in the lower density (100/m^2^) when compared with the density of 300/m^2^ as shown in Table 3. Fleckenstein et al., [35] reared shrimp at two stocking densities for 120 days and observed greater final weight and higher growth rate in the low-density group (100 shrimp/m^3^) than in the high-density group (200 shrimp/m^3^). Similarly, final shrimp weight was reported to be better in the lowest-density group (300 org./m^3^) than in the other two higher stocking densities (400 and 500 org./m^3^) [17]. Likewise, Krummenauer et al. [36] found that increased stocking densities lead to a decreased growth performance within stocking densities of 150, 300, and 450 shrimp/m^2^ for a rearing period of 120-day in indoor tanks. In addition, a better survival rate was associated with the lower density group (Table 3). Similarly, Freitas et al. [37] and Otoshi et al. [20] reported that a significantly higher survival rate was obtained at 200 shrimp/m^2^ density than 400 shrimps/m^2^ density. Reduced survival rate at the higher densities might be due to negative behavioral interactions, for example, cannibalism [38].

Total biomass was significantly increased in the higher density system. It was recorded as 97.06 ± 0.52 kg as compared with 45.77 ± 0.43 kg in the 100 org./m^2^ treatment (Table 3). Similar results were reported by Fleckenstein et al., [35] who found that total shrimp biomass production was significantly higher in high-density treatments compared to the lower densities (*P* < 0.05). Additionally, between three stocking densities of 400, 500, and 600 shrimp/m^2^, higher density supported better-harvested biomass [39].

#### 3.1.1. Feed Utilization

Lower density supported better feed utilization. It was associated with significantly higher FE (0.71 ± 0.006) and PER (1.87 ± 0.017) ratios (Table 4). The results revealed an acceptable FCR ratio in both densities which might be due to the advantage of the all-day food availability offered in the biofloc system. The natural productivity of the biofloc system could be very effective in providing the shrimp with their nutritional requirements which can help to obtain lower FCR [40, 41]. Significantly lower FCR was noted in the lower density group (1.41 ± 0.01 vs. 1.61 ± 0.01). The superior FCR ratio in the lower stocking density revealed by the results of this study is compatible with Liu et al. [17] and Tao et al. [22]. The lower feed utilization efficiency in the higher densities may be due to the stressful conditions in the high densities (crowding, lower water quality, etc.) [23, 42].

#### 3.1.2. Water Quality

All the water quality parameters in the present study were kept in the suitable ranges for shrimp culture indicated by [43–45]. Water quality parameters were all more optimized with the lower density system (Table 5). The dissolved oxygen concentration was significantly higher (*P* < 0.05) in the lower density group (5.63 ± 0.06 mg/l). As well as, decreased NH_3_ (0.023 ± 0.002), NO_2_ (0.36 ± 0.02), and turbidity (45.84 ± 1.19) were recorded in the 100 org./m^2^ treatment.

Lower DO concentration and higher ammonia and nitrite levels were observed in the higher density system which is similar to results observed by Fleckenstein et al. [35]. Higher mean nitrite concentrations in the high-density treatment (200/m^2^) were found compared to the low-density treatment (100/m^2^). Dissolved oxygen concentrations over the study period were significantly lower in the higher density treatment, likely due to increased respiration rates in the water column. It is perceptible that the culture stocking density may have a bad impact on the culture water quality, as increasing DO consumption, or increasing ammonia and nitrite levels can cause a suppression of the growth performance [36].

Increased nitrite and ammonia concentrations found in the higher density treatment were rather mutual in biofloc shrimp systems and have been detected in other studies Esparza-Leal et al., [46]. TAN has a positive relationship to rearing density, i.e., the higher the density the higher recorded TAN as nitrogen released from shrimps and uneaten feeds leftover, consequently [22]. The higher levels of nitrite and ammonia in (300 org./m^2^) stocking density compared to lower density may be related to the lower values of dissolved oxygen recorded in the high-density group [37].

Numerical lower pH (6.96 ± 0.06 vs. 7.16 ± 0.07) which was observed in the higher stocking density was also reported by Fleckenstein et al. [35], pH values throughout his study were significantly lower in high-density treatments. In contrast, Schveitzer et al., [21] observed a slightly higher pH in the higher density (473/m^3^) treatment over the lower one (238/m^3^). The lower pH and dissolved oxygen levels in the higher stocking density indicate an increased respiration rate of microbes, along with higher levels of shrimp respiration and added carbon dioxide to the water column [47, 48].

Significantly higher turbidity observed in the 300 org./m^2^ treatment (50.29 ± 1.57) may be explained by the evidence of the increased abundance of the microbial communities which probably augmented water column respiration. Floc volume was numerically increased in higher stocking density treatment (19.60 ± 0.42 vs. 19.07 ± 0.20). These results were compatible with Avnimelech [49] who reported a closer floc volume as it ranged from 20 to 25 ml/L. Tao et al. [22] also observed a higher floc volume in the density of 300/m^2^ compared to 150 and 200/m^2^ with a significant difference between the higher density and the other two densities.

### 3.2. Bacteriology

**Figure 1:**
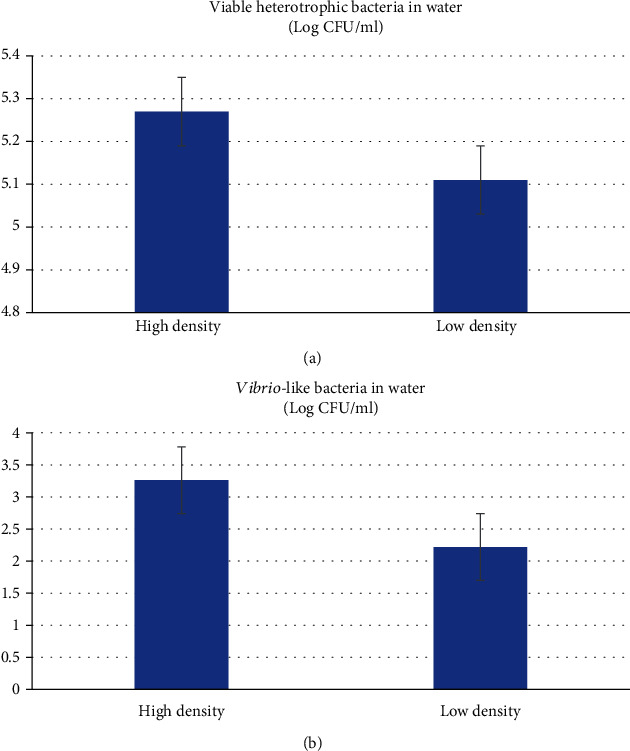
Viable heterotrophic bacteria count (a), and *Vibrio*-like bacteria count (b) (Log CFU/ml) from water collected from whiteleg shrimp (*L. Vannamei*) biofloc tanks under high (300/m^2^) and low (100/m^2^) stocking densities.

Heterotrophic bacterial count (HBC) in water samples from high-density treatment (300/m^2^) was 5.28 ± 0.15 log CFU/ml, while their count in lower density system was 5.11 ± 0.28 log CFU/ml, with no significant differences (*P* > 0.05) as results show (Figure 1(a)). Heterotrophic bacterial load in water samples were recorded in the biofloc system at densities of 500 in./m at day 30 ranging from 38.2 to 65.3 × 10^6^ CFU/ml [50]. Similarly, the values of total heterotrophic bacteria count were recorded as 225.78, 178.68, and 341.18 × 10^5^ CFU/ml in 12, 14, and16 larvae/l for 90 days, respectively [51]. The salinity of seawater might decline the bacterial load. A reverse correlation was previously recorded between the level of indicator bacteria such as HBC and the salinity of seawater samples [52].

*Vibrio* spp. were known as heterotrophic bacteria that efficiently utilize carbohydrates present in the water [53]. In this study, *Vibrio*-like count in water samples were higher (*P* < 0.05) in high-density system (3.26 ± 0.23 log CFU/ml) compared to low-density system (2.22 ± 0.5 log CFU/ml) (Figure 1(b)). Tao et al. [54] indicated *Vibrio* concentration in the rearing medium recorded as 30.83 × 10^3^ CFU/ml did not harm the tiger shrimp (*Penaeus monodon)* in the hatchery. Arias-Moscosoa et al. [50] indicated similar results in shrimp biofloc at day 30 as 1.67-4.23 × 10^3^ CFU/ml at densities of 500 in./m even with the addition of commercial probiotics shrimp farm.

*Bacillus* spp. were identified in water samples from both high- and low-density systems. *Bacillus* spp. such as *B. subtilis* are considered beneficial bacteria that biofloc system targeted its growth and multiplication. It can improve the water quality, as well as promote the health of cultured shrimp [5]. *Bacillus subtilis* displayed antibacterial activities against Gram-positive and Gram-negative such as *Vibrio* spp. This might explain the absence of pathogenic *Vibrio* spp. in water and shrimp samples in both densities in this study. Likewise, Zhao et al. [4] described that adding *Bacillus* into biofloc water resulted in a decrease in *Vibrio* abundance.

Higher density system was associated with *Cronobacter* spp bacteria in water samples that were identified using API 20E diagnostic strips (Table 6). Species of *Cronobacter* such as *sakazakii* are considered foodborne pathogens [55], which might reflect on food bacterial quality in high-density systems. Other bacteria such as *Enterobacter cloacae* were isolated in water systems of both densities (Table 6). *Enterobacter* spp. is a natural commensal of the animal gastrointestinal tracts microbiota [56]. It might occur in water samples in the high- and low-density systems in this study due to the accretion of shrimp commensals as biofloc are characterized by a zero-water exchange closed system. *Enterobacter cloacae* might be helpful in aquaculture water treatment and recorded with denitrification abilities [57]. The efficiencies of inorganic nitrogen removal of *E. cloacae* were 72.27 to 96.44%. This bacterium was also described as a probiotic supplement that advanced weight gain and controlled fish diseases such as yersiniosis [58]. *Enterobacter* was found to comply with seawater salinity (20 PSU) [59], while in this study it was associated with salinity up to 30 ± 0.5 ppt.

**Figure 2:**
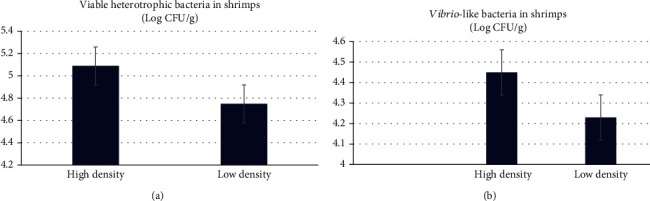
Viable heterotrophic bacteria count (a), and *Vibrio*-like bacteria count (b) (Log CFU/g) in shrimps (*L. Vannamei*) reared under biofloc system in high (300/m^2^) and low (100/m^2^) stocking densities.

The bacterial food quality of shrimps reared in the biofloc system is a challenge, due to the possibility of the presence of pathogenic microorganisms that might cause foodborne diseases. In this study, shrimp samples were selected and bacteriologically analyzed to inspect the reflection of water quality and different densities on its bacterial quality. Total bacterial count in this study was recorded as 5.09 ± 0.1 log CFU/g in high-density system compared to 4.75 ± 0.24 log CFU/g (*P* < 0.05) (Figure 2(a)). Still, the total number did not exceed the maximum level of the Egyptian Organization for Standardization and Quality Control [60] of fresh chilled shrimp (less than 10^6^ CFU/g). In the same way, no statistically significant differences (*P* > 0.05) were recorded between the *Vibrio*-like bacterial count from shrimp samples of the high-density system (4.45 ± 0.25 log CFU/g) compared to the low-density system (4.23 ± 0.32 log CFU/g) (Figure 2(b)). *Escherichia coli* were identified from shrimp samples in a low-density biofloc system (Table 6), but its pathogenicity was not detected. The presence of *E. coli* in shrimp is probably due to fecal pollution of the environment [61].

Pathogenic bacteria that were identified in shrimp samples in a higher density biofloc system were *Aeromonas hydrophila* and *Citrobacter freundii* (Table 6). *Aeromonas hydrophila* is reported as a foodborne pathogen causing human gastroenteritis that inhabitants in the aquatic environment [62]. It was implicated in numerous outbreaks of seafood poisoning in different countries [62, 63]. Generally, *A. hydrophila* is not considered to be a marine bacterium [64]; however, other studies indicated that it is found naturally in marine systems, and it can be found at all salinities, except the most extreme (>100%) [65, 66]. *Citrobacter freundii* belongs to the family Enterobacteriaceae which is a normal inhabitant in the gastrointestinal tract of animals and humans and causes foodborne intoxication [17, 67, 68]. Enterobacteriaceae documented accounted for almost 20% of the bacterial communities of shrimp cultured in seawater BFT system [69]. Though, neither the virulence genes of pathogenicity of the previous bacteria nor their effects on human health were detected in this study.

High-density biofloc system suffered the stress of crowdies that may decrease shrimp immunity, which in return leads to a higher susceptibility of the shrimps to bacterial infections. Liu et al. [17] documented that resistance to *Vibrio harveyi* infection can be weakened due to high density in the biofloc system. Proper handling of shrimp especially in high-density biofloc systems and good hygienic measures were necessary to inhibit any potential risk of foodborne infection.

### 3.3. Gene Expression

#### 3.3.1. Immune, Stress, and Growth-Related Genes Expression

Growing shrimp in a biofloc system is considered an alternative strategy to improve the environmental conditions and health status of cultured animals. Biofloc can act as immuno-stimulants to improve the shrimp's native immune system and provide protection against bacterial pathogens [16]. The innate immune system of shrimp is composed primarily of phagocytosis, microbial recognition systems, prophenoloxidase (proPO) activating system, clotting system, encapsulation, nodule formation, antimicrobial proteins, and reactive oxygen compounds [70]. A dense microbial population in biofloc systems may activate the nonspecific shrimp immune system, resulting in a type of defense that may permit a quick response against bacterial diseases [17]. It was distinguished that shrimps grown in the biofloc system concealed a higher total antioxidant capacity [71]. The awareness of suitable stocking density for the production of whiteleg shrimp in the BFT system is an effective approach to avoiding stocking density's bad impacts on shrimp growth and health status.

**Figure 3:**
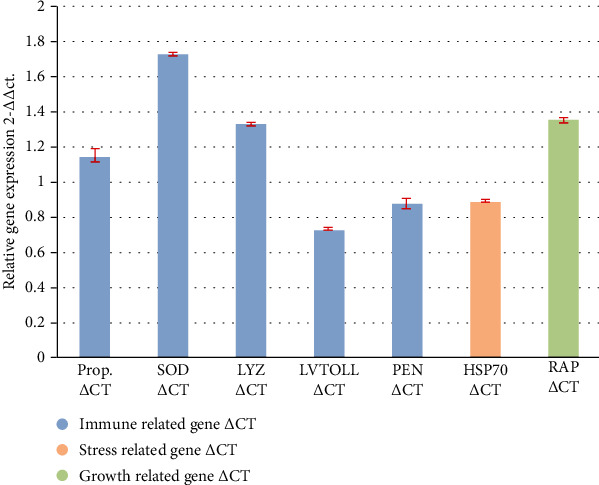
Relative expression levels (mean ± SD) of *P. vannamei* hemolymph immune, stress, and growth-related genes after a 135-days rearing period under a density of 100 org./m^2^ as compared to the control group (300 org./m^2^).

Results revealed that prophenoloxidase (proPO) gene expression of shrimps from the lower density treatment was significantly (*P* < 0.05) higher expressed than that of the higher density group. Additionally, this increased expression was also observed in other immune-related genes such as superoxide dismutase (SOD) and lysozyme (LYZ) (Table 7 and Figure 3).

Lysozyme is a prominent antimicrobial peptide (AMP) that directly began an assault against pervading pathogens by enhancing the hydrolysis of the cell wall of invading bacteria [72]. In shrimp, lysozyme was found to display antimicrobial activity against both Gram-negative and Gram-positive bacteria including *Vibrio* species that are pathogenic to shrimp [73]. Immuno-stimulants can elevate the LYZ activity by triggering the amount of LYZ produced by the cell [74].

During respiration, shrimps synthesized reactive oxygen radicals (ROS) such as peroxide, superoxide, and hydroxyl ion to kill the pathogens. Shrimps protect themselves from the lethal effect of ROS by secreting oxidative enzymes like catalase and SOD. The SOD is the main antioxidant enzyme linked to immunity in crustaceans. Many studies claimed that SOD activity in the hemolymph of BFT-reared shrimp was of higher levels [75]. The immune status of whiteleg shrimp could be impaired in the high-density conditions in biofloc systems [17]. The above finding was also supported by Lin et al. [76], who stated that high densities showed low resistance against pathogens assigned by a decrease in immune parameters with declined expression levels of immune-related genes.

As for stress-related genes, results (Table 7 and Figure 3) showed that the expression of TOLL receptor and HSP70 genes were upregulated in the higher density group with significant differences (*P* < 0.05). Toll-like receptors (TLRs) are identified as quite preserved proteins across the cell membrane of immune cells. TLRs respond quickly to PMAPs (pathogen-associated molecular patterns) of microorganisms (lipopolysaccharides, peptidoglycans, and *β-*glycans) [77]. TLRs are attributed to proinflammatory cytokines, and chemokine production boosts antimicrobial responses [78] [79]. In this study, the upregulation was an obvious response of Toll gene expression in the higher density group accompanied by the upregulation of HSP70. This finding parallels the finding of Gárate et al. [80]. TLRs expression is regulated in response to environmental stressors.

Heat shock proteins (HSP) are much conserved proteins well known for their quick responses to stress [81]. With the existence of environmental stresses, Hsp70s work to ease the degradation of irreversibly-denatured proteins, inhibit protein aggregation, and repair partially-denatured proteins [82]. HSP70 amplified in hepatopancreas with growing stocking density of *Litopenaeus vannamei* resulting in decreased stress resistance capacity [83]. Hsp70s are also implicated in eliciting immune responses against many bacterial diseases [84].

Probiotics interconnect with the host by pattern recognition receptors (PAMP) such as nucleotide-binding oligomerization domain-containing protein-like receptors and toll-like receptors. Therefore, it modulated signaling pathways such as nuclear factor-*κ*B to increase or decrease activation and influence downstream pathways. This recognition is vital for producing measured-antimicrobial responses with slight inflammatory tissue damage [85]. This finding was in harmony with the result of PEN4 in this study where its regulation was significantly decreased in the lower density-raised group (*P* < 0.05).

The expression of the Rap-2a gene (growth-related gene) was significantly downregulated in the higher density treatment that was supposed to stress density. Rap-2a is a member of the Ras-related protein family and a part of several signaling cascades. It might regulate cell migration, cell spreading, and cytoskeletal rearrangements [86]. Growth reduction, in general, is considered to be a good indicator of chronic stress, and in some species, density acts as a chronic stressor. Several studies reported decreased growth associated with increasing density [17]. Downregulation in higher density group Rap-2a may be considered as a body response to avoid an unbalanced immune response as overexpression of Rap-2a was reported to lead to severe inhibition of NF-*κ*B activation and subsequent TLR signaling molecules.

## 4. Conclusions

Stocking density has many effects on the production of whiteleg shrimp under the biofloc system. Final weight, weight gain, daily weight gain, specific growth rate, biomass increase percentage, and survival rate were all significantly higher (*P* < 0.05) in a stocking density of 100 org./m^2^ as compared with the 300 org./m^2^ system. Significantly higher feed efficiency (0.71 ± 0.006) and protein efficiency ratio (1.87 ± 0.017) were found with the lower density treatment. A better feed conversion ratio was noted in the lower density group **(**1.41 ± 0.01 vs. 1.61 ± 0.01). Dissolved oxygen concentration was significantly higher (*P* < 0.05) in the lower density group (5.63 ± 0.06 mg/l) while NH_3_ (0.023 ± 0.002) and NO_2_ (0.36 ± 0.02) were both significantly lower than of those at 300 org./m^2^ (0.032 ± 0.001 and 0.52 ± 0.02, respectively). Beneficial bacteria such as *Bacillus* spp. and *cloacae* were isolated in water samples from both high- and low-density systems. The total bacterial count in shrimp was recorded as 5.09 ± 0.1 log CFU/g in the 300 org./m^2^ treatment compared to 4.75 ± 0.24 log CFU/g in the lower density. Pathogenic bacteria were related to the higher density system, such as *Cronobacter* spp from water, *Aeromonas hydrophila*, and *Citrobacter freundii* from shrimp. Immune-related genes including prophenoloxidase, superoxide dismutase (SOD), and lysozyme (LYZ) expressions were all higher in the shrimps from the 100 org./m^2^ treatment. Toll receptor (LvToll), penaiedin4 (PEN4), and HSP 70 genes showed a significant decrease in their expression in shrimps that raised under the lower stocking density. *S*ignificant upregulation of Ras-related protein (Rap-2a) expression was associated with the lower stocking density group. Generally, practicing of ultraintensive shrimp biofloc system (300 org./m^2^) did not contribute desirable effects on performance, water quality, microbial community, bacterial food quality, and gene expression when compared with the lower density system (100 org./m^2^).

## Figures and Tables

**Table 1:** Proximate analysis of experimental feed and carbon sources used in the study.

Constituent	Feed	Wheat flour
Crude protein	38.20	10.38
Ether extract	10.27	3.32
Crude fiber	4.75	5.28
Total ash	8.95	1.68
Moisture	7.98	10.91
Nitrogen free extract	29.85	68.43

Values are on a dry weight basis.

**Table 2:** Sequences of primers submitted for RT-qPCR to assess immunological, stress, and growth state of shrimp, *L. vannamie.*

Immune element/stress response and growth	Gene	Abbreviation	PrimerF/R	(5^`^-3̀) primer sequence	References
proPO activating system	Prophenoloxidase	proPO	proPO-F	GAG ATC GCA AGG GAG AAC TG	([77, 87, 88])
			proPO-R	CGT CAG TGA AGT CGA GAC CA	

Antimicrobial peptide system	Lysozyme	Lyz	Lyz-F	GAA GCG ACT ACG GCA AGA AC	([87, 88])
			Lyz-R	AAC CGT GAG ACC AGC ACT CT	
	Penaiedin4	PEN4	PEN4-F	GCC CGT TAC CCA AAC CAT C	([87, 89])
			PEN4-R	CCG TAT CTG AAG CAG CAA AGT C	

Antioxidant defense mechanism	Superoxidase dismutase	SOD	SOD-F	ATC CAC CAC ACA AAG CAT CA	([77, 87–89])
			SOD-R	AGC TCT CGT CAA TGG CTT GT	

Pattern recognition receptor	Toll receptor	LvToll	LvToll-F	ATG TGC GTG CGG ATA CAT TA	([77, 87, 88])
			LvToll-R	GGG TGT TGG ATG TCG AGA GT	

Stress	Heat shock protein 70 (Lvhsp70)	HSP70	hsp70 -F	GGC AAG GAG CTG AAC AAG TC	([88, 89])
			hsp70 -R	TCT CGA TAC CCA GGG ACA AG	

Growth	Ras-related protein rap-2a	Rap-2a	RAP-2a-F	GCC GTG CGT GCT TGA GAT	[86]
			RAP-2a-R	TTG ATG TCC TGG AAG GTC TGG	

Internal control	*β*-Actin		*β*-Actin-F	CCA CGA GAC CAC CTA CAA C	([77, 89, 90])
			*β*-Actin-R	AGC GAG GGC AGT GAT TTC	

**Table 3:** Growth performance parameters and survival rate of whiteleg shrimp *L. vannamei* reared under different density biofloc systems for 135 days.

	Stocking density		Sig.
	100/m^2^	300/m^2^	
Final weight (g)	15.36 ± 0.05	11.27 ± 0.03	0.000
Weight gain (g)	15.33 ± 0.05	11.24 ± 0.03	0.000
ADG (g)	0.137 ± 0.00	0.100 ± 0.00	0.000
SGR %	5.57 ± 0.00	5.29 ± 0.00	0.000
Biomass (kg)	45.77 ± 0.43	97.06 ± 0.52	0.000
Biomass increase %	509.43 ± 2.16	359.38 ± 3.56	0.000
Survival rate%	99.33 ± 0.38	95.7 ± 1.16	0.042

**Table 4:** Feed utilization parameters for whiteleg shrimp *L. Vannamei* reared under different density biofloc systems for 135 days.

	Stocking density		Significance
	100/m^2^	300 /m^2^	
FCR	1.41 ± 0.01	1.61 ± 0.01	0.001
FE	0.71 ± 0.00	0.62 ± 0.00	0.001
PER	1.87 ± 0.01	1.63 ± 0.01	0.001

**Table 5:** Water quality parameters during 135 days rearing period of whiteleg shrimp under different density biofloc systems.

	Stocking density		Sig.
	100/m^2^	300/m^2^	
DO	5.63 ± 0.06	5.06 ± 0.00	0.000
NH3	0.02 ± 0.00	0.03 ± 0.00	0.006
NO2	0.36 ± 0.02	0.52 ± 0.02	0.000
pH	7.16 ± 0.07	6.96 ± 0.06	0.053
Turbidity	45.84 ± 1.19	50.29 ± 1.57	0.033
Biofloc volume	19.07 ± 0.20	19.60 ± 0.42	0.27

**Table 6:** Biochemical test results of different isolated bacteria using API 20E diagnostic strips from shrimp and tanks under biofloc system under high (300/m^2^) and lower (100/m^2^) stocking density.

	Stocking density					
	300/m^2^				100/m^2^	
	Water		Shrimp		Water	Shrimp
	*Cronobacter* spp.	*Enterobacter cloacae*	*Citrobacter freundii*	*Aeromonas Hydrophila*	*Enterobacter cloacae*	*E. coli*
ONPG	+	+	+	+	+	+
Arginine dihydrolase	+	+	+	+	+	+
Lysine decarboxylase	—	—	—	+	—	+
Ornithine decarboxylase	+	+	+	—	+	—
Citrate	+	+	+	—	+	—
H_2_S	—	—	+	—	—	—
Urease	—	—	—	—	—	—
TDA	+	+	+	—	—	—
Indole	—	—	—	—	—	+
Voges-Proskauer	—	+	—	+	+	—
Gelatinase	+	—	+	+	—	—
Acid from:						
Glucose	+	+	+	+	+	+
Mannitol	+	+	+	+	+	+
Inositol	—	—	—	—	—	—
Sorbitol	+	+	+	—	+	+
Rhamnose	+	+	+	—	+	+
Sucrose	+	+	+	+	+	+
Melibiose	+	+	+	—	+	+
Amylose	—	+	+	—	+	—
Arabinose	+	+	+	—	+	+

**Table 7:** Fold change in expression levels (mean ± SD) of tested genes for white leg shrimp *L. Vannamei* reared under stocking density of 100 org. /m^2^ under biofloc system compared to of higher density (300 org./m^2^) control group.

Gene	Fold change ± SD
Prophenoloxidase	1.15 ± 0.07
Lysozyme	1.33 ± 0.01
Penaiedin4	0.88 ± 0.05
Superoxidase dismutase	1.73 ± 0.02
Toll receptor	0.73 ± 0.01
Heat shock protein 70 (Lvhsp70)	0.89 ± 0.01
Ras-related protein rap-2a	1.35 ± 0.02

## Data Availability

The research data associated with a paper is available, and the data can be accessed.
